# Validation of the Hungarian Version of the COVID Stress Scale (CSS-H)

**DOI:** 10.3390/ijerph191912602

**Published:** 2022-10-02

**Authors:** Ágnes Pálvölgyi, Kata Morvay-Sey, Pongrác Ács, Dávid Paár

**Affiliations:** 1 Doctoral School of Health Sciences, Faculty of Health Sciences, University of Pécs, Vörösmarty u.4., 7621 Pécs, Hungary; 2 Institute of Physiotherapy and Sport Sciences, Faculty of Health Sciences, University of Pécs, Vörösmarty u.3., 7621 Pécs, Hungary

**Keywords:** Hungarian COVID Stress Scale (CSS-H), pandemics, fear, anxiety, COVID-19

## Abstract

(1) Background: The COVID-19 pandemic is unprecedented and has affected every social class. The prevalence of moderate-to-severe stress and anxiety levels in the general population was reported to be 25%. This study aimed to describe the validation of the Hungarian version of the COVID Stress Scale. (2) Methods: The research study was a cross-section validation study with a representative sample (N = 1200) and a non-representative student sample (N = 350). The translation procedure was a four-step procedure. The interviewers conducted the data collection. (3) Results: The reliability of the Hungarian CSS was assessed using Cronbach’s alpha. Convergent validity was evaluated by correlating the CSS with the PSS and WHO-WBI5. The Cronbach’s alpha coefficient of the CSS-H factors was between 0.844 and 0.907 (representative sample) and between 0.878 and 0.936 (student sample), which qualified as very good. The reliability of the internal consistency was good for all six factors in both samples. The CSS-H total and all-domain scores significantly and positively correlated with the PSS total score and negatively correlated with well-being. (4) Conclusions: The Hungarian COVID Stress Scale is a valid, reliable instrument to measure COVID-19-related distress in the Hungarian population.

## 1. Introduction

The 2019 coronavirus (COVID-19) pandemic has been one of the most extensive in human history. The pandemic situation has been unprecedented and has affected all layers of society. The pandemic disrupted the healthcare system, economic stability, social functioning, and daily life. The virus reached Hungary in early March 2020 [1], and the Hungarian government announced a total lockdown that lasted from 28 March 2020 to 4 May 2020 [2]. After the summer, the second wave reached Hungary in November 2020. The government reinstated the special rule of law, announced a health emergency, and reimposed restrictions on 4 November 2020 [3]. No vaccinations were available during these first two waves, and the number of deaths rose. The virus variants (Delta, Omicron) posed significant challenges during the third, fourth, and fifth waves. Vaccination offered only partial protection against the virus, but it did attenuate the adverse reactions, which was relieving. Nevertheless, Hungarians were far from safe.

Previous studies demonstrated that the general population experienced severe-to-moderate anxiety during other pandemics (2009 H1N1, SARS outbreak) [4,5]. In 2020, data from China were similar to data from these previous pandemics. Researchers showed that 25% of the general population experienced moderate-to-severe stress and anxiety levels [6,7,8]. Concerning stress, fear (fear of becoming infected, fear of contaminating, etc.) and anxiety-guided behavior (hand washing, distancing, compulsive checking, etc.) are usual in such situations. The mental health consequences of the COVID-19 pandemic are traumatic and are similar to those experienced in natural disasters and terrorist attacks [9,10]. Poor sleep quality [11,12], depression symptoms [13], and general anxiety disorder all increased [14,15]. Some research studies connected to mental health status were conducted in Hungary during the COVID-19 pandemic. Szabó et al. [16] measured stress with the Perceived Stress Scale (PSS-10) and found that the mean of the participants’ perceived stress scores was 19.34 ± 7.97, which was higher than the normal mean (17.5 ± 6.0) (Csikós et al. (2020)) [17] measured 346 parent–child dyads. They used the PSS and found the same as above. The level of perceived stress in adults was higher than expected in a normal population; it was closer to the clinical population’s level [18]. Morvay-Sey et al. (2022) [19] discovered the same in a university student sample; PSS-14 was used during the first wave of the pandemic. The perceived stress total score in this survey was 31.4 ± 10.35, which was much higher than the normal mean (25.4 ± 7.8) and closer to the mean of the clinical population (30.3 ± 8.1–34.7 ± 8.09). Rucska-Lakatos [20] investigated the population above the age of 18 in the northeastern region of Hungary during the second wave of the pandemic. Their results proved COVID-19’s negative effect on mental health with increases in depression symptoms, anxiety, and perceived stress. These results correlated with the national results. Based on clinical observation, Taylor et al. (2020a) [21] suggested that COVID-19 Stress Syndrome is multifactorial—a complex phenomenon involving various fears. The most significant factor is the fear accompanying the perceived danger associated with the virus. COVID-19 Stress Syndrome involves xenophobia, traumatic stress symptoms, compulsive checking, and reassurance seeking. Other variables connected to the phenomenon include panic buying and maladaptive coping. COVID- Stress Syndrome could be its own disorder, or it might be a part of another disorder (e.g., GAD, OCD, PTSD). Taylor et al. (2020b) [22] developed a 36-item COVID Stress Scale (CSS) to understand and measure COVID-19-related distress. The goal was to adapt the scale for future pandemics. The instrument has five factors: (1) danger and contamination fears; (2) fears about economic consequences, (3) xenophobia; (4) compulsive checking and reassurance seeking; (5) traumatic stress symptoms about COVID-19. Each scale includes six items, and the scales tend to be strongly intercorrelated. This intercorrelation is essential to providing evidence for the existence of a coherent syndrome. The intercorrelation was first developed using a representative sample from Canada (N = 3479) and was deemed valid and reliable. Afterwards, Taylor et al.(2020b) [22] used it on a sample from the United States (N = 3375). Items are rated on a five-point Likert scale, where 0 = not at all and = 4 extremely. The scale demonstrates good-to-excellent internal-consistency reliability.

The English version of the CSS, which was originally developed for English-speaking North American samples, had not been translated into Hungarian before (it has already been translated into several languages (https://coronaphobia.org/ accessed on 24 September 2022)). The CSS authors permitted our research group to translate, adapt, and validate the COVID Stress Scale in Hungary. This study aimed to measure the psychometric characteristics (confirmatory factor analysis, convergent validity) of the Hungarian version of the CSS.

The second wave of the COVID-19 pandemic hit the Hungarian population severely. Infection numbers and mortality ratios increased exponentially. Understanding interpersonal variables such as fear, stress, anxiety, the related behavior, and the network of these factors is vital. By applying the knowledge gathered, we can determine treatment methods. The validation of the CSS aims to better understand COVID-19 Stress Syndrome.

## 2. Materials and Methods

The research study was a cross-section validation study conducted from 7 December 2020 to 13 December 2020 (population sample) and from 9 December to 23 December 2020 (student sample). Developing the CSS-H was a multistep process. The first step was to have two independent translators translate the English version into Hungarian. The second step involved comparing the two translations to establish a conventional version. The third step entailed translating the conventional version back into English to verify the accuracy of the translation. The final version used by an interviewer comprised the fourth step (Table A1).

Concerning the data collection in the population sample, of the 60 interviewers who participated in the research study, 7 were experts with more than 10 years of interviewing experience. The interviewers received all the instructions about the survey before the data collection commenced. The recruitment was face-to-face. The interviewer wore a mask and maintained a 1.5 m distance during the interview. After every interview, interviewers disinfected their equipment and hands. To ensure data quality, every interviewer had a maximum of 36 questionnaires. Since data quality was vital during data collection, we reviewed all interviews the following day. The circumstances and duration of the interview were determined while checking the demographic variables. The mean duration of the interviews was 25 min 17 s ± 5 min 29 s.

The first sample (n = 1200) was a nationally representative sample of Hungary’s population by gender, region, education, and residence type covering the age group of 16–69 years.

Data collection in the student sample: The sample was conducted at University of Pécs, Hungary. All the respondents were adults and active students (BSc, MSc, PhD, and postgraduate) in one of the 10 faculties of University of Pécs. The data were obtained through a web-based comprehensive questionnaire method (with a free Google account—Google Forms), using the snowball method, for convenience sampling. Participants were informed about the research aim and methods before signing the informed consent form. The investigation conformed to the principles outlined in the Declaration of Helsinki. The second sample (n = 350) was not considered to be representative.

The validation process was performed on these two samples. Table 1 summarizes the composition of the samples.

Taylor et al. (2020b) [22] used an exploratory factor analysis (EFA) with the robust maximum likelihood (RML) method with oblimin rotation to determine the factor structure of their English version questionnaire containing 36 items. The items were sorted into five factors, despite the assumption of six relevant factors including 6–6 questions per factor. The “danger” and “contamination” factors were grouped into one factor. Taylor et al. also ran a confirmatory factor analysis (CFA) using RML estimation, which confirmed the 5-factor structure. The same 5-factor structure was also used to validate the questionnaire in Arabic [23], Persian [24], and Greek [25]. The Serbian [26] and Spanish (Peru) versions [27] have 6-factor structures.

However, since all 36 questionnaire items are ordinal Likert-scale items that do not follow a normal distribution, it was more optimal to use diagonally weighted least squares (DWLS) estimation based on the literature recommendations [28,29]. Due to the change in the estimation method compared with Taylor et al. (2020b) [22], the confirmatory factor analysis (CFA) was run with not only the 5-factor structure validated by Taylor et al. (2020b) [22] but also with the 6-factor structure they originally assumed.

Confirmatory factor analyses were run on two independent samples (a representative national sample and a non-representative student sample), using all 36 original questionnaire queries.

To process the confirmatory factor analysis and to check the internal consistency of the resulting factors, we used JASP software version 0.14.1 (JASP Team, 2020) [30].

To evaluate the confirmatory factor analysis, we used the following fit indicators (their expected cut-off values are in parentheses) based on Hu–Bentler (1999) [31]: comparative fit index (CFI; 0.95), Tucker–Lewis index (TLI; 0.95), standardized root mean square residual (SRMR; 0.08), root mean square error of approximation (RMSEA; 0.06).

The internal consistency of the resulting scales was measured using Cronbach’s alpha, with an acceptance range of 0.70 to 0.95 [32].

The convergent validity of the CSS questionnaire was tested using the Perceived Stress Scale (PSS-14) and the WHO Well-Being Inventory (WBI-5). Spearman’s correlation coefficient was used to test this.

### Perceived Stress Scale (PSS-14)

We used the validated 14-item Hungarian version of the Perceived Stress Scale (PSS) [17,31]. The questionnaire was measured on a 5-point Likert scale (0–4), where 0 meant “never” and 4 meant “very often”. The scale had good internal reliability (Cronbach’s alpha 0.88) and a good test–retest value (r = 0.90). The questionnaire asked how much stress the person had experienced in the past month (subjectively) and how unpredictable, uncontrollable, and overloaded he/she considers everyday life to be.

#### WHO Well-Being Inventory Short Version (WBI-5)

The WHO Well-Being Inventory measures the positive quality of life, for which the 5-item (WBI-5) abbreviated Hungarian version [33] was used. Respondents scored the statements on a four-point Likert scale, in which 0 signified “no time” and 3 indicated “all of the time”. The internal reliability of the questionnaire was excellent (Cronbach’s alpha: 0.85).

The Hungarian Scientific and Research Ethics Committee (TUKEB IV/4599-2/2020/EKU) and Regional Research Ethics Committee (REKEB) granted the ethical approval for the study. Participants were informed about the research aim and methods before signing the informed consent form. The investigation conformed to the principles outlined in the Declaration of Helsinki.

## 3. Results

### Confirmatory Factor Analysis (CFA), Internal Reliability, and Validity

The confirmatory factor analysis conducted on the two samples was a good fit for the five-factor (validated by Taylor et al., 2020b [22]) and the six-factor cases. Only the five-factor model of the representative sample had an SRMR index above the threshold (Table 2). Both model specifications of the representative sample resulted in high and significant χ2 values due to the large sample size. After comparing the goodness-of-fit indicators for the five- and six-factor models, we believe that the use of the six-factor structure originally envisioned by Taylor et al. (2020b) [22] is justified; however, it was reduced during their validation process.

Table 3 contains the factors and their items translated into Hungarian from the original questionnaire, as well as the factor loadings and Cronbach’s alpha values obtained in the confirmatory factor analysis. The internal consistency index fell within the acceptable range for all factors.

The total score of the CSS questionnaire and the total score of each factor in both samples were also compared to the total scores of the WBI-5 and PSS questionnaires using Spearman’s correlation coefficients when processing the convergent validity test (Table 4).

## 4. Discussion

The COVID-19 pandemic remains the leading acute global medical problem today. After the second wave occurred, the third, fourth, and fifth waves and additional virus variants (e.g., Omicron) followed. Hungary has offered vaccinations since 2021, but these do not provide 100% protection against the virus. So, the chance of becoming infected and the related pandemic stress remains. In addition to the somatic effects, measuring the mental and emotional effects of the COVID-19 pandemic is still important. Our study analyzed the psychometric properties of the Hungarian COVID Stress Scale (CSS-H) and demonstrated the validation process. According to our results, Hungary has experienced all six factors (danger, socio-economic consequences, xenophobia, contamination, traumatic stress, and compulsive checking). This result concorded with the Serbian [26] and Spanish (Peru) versions [27]. We also checked the convergent validity of the CSS-H with other psychological scales (PSS, WHO-WBI5) and found a significant relationship with both the WBI-5 and the PSS scales. As expected, the total scale score showed a negative relationship with the WBI-5 scale score in both samples—i.e., a high level of stress was associated with a decline in well-being. At the same time, the total score of the PSS showed a positive correlation in both samples, which was expected, as both scales measure the individual’s stress perception. Similar to the total score of the CSS-H, there were also significant negative relationships among the single factors of danger, socio-economic consequences, and traumatic stress in both samples, and between them and the WBI-5 scale for compulsive checking in the university sample. Similar to the total score, positive correlations were found in the PSS scores in both samples among danger, socio-economic consequences, and traumatic stress factors. Positive correlations appeared between the xenophobia factors in the representative sample and the contamination and compulsive checking factors in the university sample. The internal consistency of the CSS-H was good. When compared to international COVID Stress Scales in order of magnitude, it proved adequate.

## 5. Conclusions

In conclusion, the Hungarian version of the COVID Stress Scale possesses excellent psychometric features; it preserves the original psychological construct. Accordingly, it could measure attitudes toward COVID-19 among the Hungarian population in future epidemics. Mental health problems are already a significant social burden, so it is urgent to understand the relationship system and the exact background of health problems. We need appropriate tools and questionnaires to create health promotion strategies, especially in the mental health field.

This study suggests that the CSS-H in the Hungarian general population is a valid and reliable questionnaire to assess distress related to the COVID-19 pandemic. The CSS in Hungarian incorporates six domains. We recommend this questionnaire for further research about mental health during the current pandemic in Hungary.

## 6. Limitation

Due to the temporality of the event, our research study followed a cross-sectional study design. We do not have information concerning the previous conditions of the participants. Some participants may have had anxiety problems in the past. Similarly, we know nothing about the participants’ characters. The student sample had more females than males, which mirrors several other research studies. Nevertheless, the strength of our study lies in the representativeness of the population sample.

## Figures and Tables

**Table 1:** Characteristics of the samples used for validation.

	Representative Sample (n = 1200)	Sample of Students (n = 350)
Age (years)	43.37 ± 15.13	25.36 ± 8.48
**Gender**		
Male	580 (48.3%)	64 (18.2%)
Female	620 (51.7%)	287 (81.8%)
**Age category**		
Age category 18–29	276 (23.0%)	
Age category 30–39	209 (17.4%)	
Age category 40–49	305 (25.4%)	
Age category 50–59	193 (16.1%)	
Age category 60–69	217 (18.1%)	
**Marital status**		
Single	410 (34.2%)	204 (58.1%)
Living with a partner	262 (21.8%)	103 (29.3%)
Married	510 (42.5%)	44 (12.5%)
Widow	8 (0.7%)	
Other	10 (0.8%)	
**Educational level**		
Primary	243 (20.3%)	
Skilled worker	320 (26.7%)	
Secondary	382 (31.8%)	
University degree	255 (21.3%)	
**Settlement type**		
Budapest	214 (17.8%)	25 (7.1%)
County seat	196 (29.4%)	140 (39.9%)
Other city	437 (36.4%)	107 (30.5%)
Village	353 (29.4%)	79 (22.5%)

**Table 2:** Goodness-of-fit indicators for the 5-factor and 6-factor models of confirmatory factor analyses run on representative and student samples.

	Representative Sample (n = 1200)		Student Sample (n = 350)	
	5-Factor Model	6-Factor Model	5-Factor Model	6-Factor Model
χ^2^	3442.289 ***	2879.785 ***	563.627	397.409
CFI (≤0.950)	0.947	0.958	1.000	1.000
TLI (≤0.950)	0.943	0.954	1.002	1.015
SRMR (≤0.080)	0.087	0.080	0.070	0.060
RMSEA (≤0.060)	0.064	0.058	0.000	0.000

*** *p* < 0.01.

**Table 3:** Factor loadings of the items of the Hungarian validated 6-factor questionnaire in the confirmatory factor analysis and Cronbach’s alpha values of each factor.

	Question	Representative Sample (n = 1200)		Student Sample (n = 350)	
		Factor Loading	Cronbach-Alpha ^1^	Factor Loading	Cronbach-Alpha
**Danger**			**0.907**		**0.878**
I am worried about catching the virus.	D1	0.713	0.909	0.692	0.876
I am worried that I can’t keep my family safe from the virus.	D2	0.798	0.888	0.930	0.86
I am worried that our healthcare system won’t be able to protect my loved ones.	D3	0.774	0.882	0.885	0.849
I am worried our healthcare system is unable to keep me safe from the virus.	D4	0.778	0.887	0.962	0.853
I am worried that basic hygiene (e.g., handwashing) is not enough to keep me safe from the virus.	D5	0.767	0.887	0.994	0.845
I am worried that social distancing is not enough to keep me safe from the virus.	D6	0.790	0.885	1.002	0.852
**Socio-Economic Consequences**			**0.911**		**0.936**
I am worried about grocery stores running out of food.	SE1	0.462	0.895	0.674	0.923
I am worried that grocery stores will close down.	SE2	0.383	0.905	0.701	0.931
I am worried about grocery stores running out of cleaning or disinfectant supplies.	SE3	0.442	0.885	0.845	0.916
I am worried about grocery stores running out of cold or flu remedies.	SE4	0.576	0.887	0.813	0.921
I am worried about grocery stores running out of water.	SE5	0.342	0.894	0.669	0.926
I am worried about pharmacies running out of prescription medicines.	SE6	0.619	0.906	0.884	0.929
**Xenophobia**			**0.880**		**0.927**
I am worried that foreigners are spreading the virus in my country.	X1	0.520	0.847	0.796	0.917
If I went to a restaurant that specialized in foreign foods, I’d be worried about catching the virus.	X2	0.563	0.881	0.565	0.923
I am worried about coming into contact with foreigners because they might have the virus.	X3	0.515	0.842	0.671	0.904
If I met a person from a foreign country, I’d be worried that they might have the virus.	X4	0.521	0.843	0.651	0.903
If I was in an elevator with a group of foreigners, I’d be worried that they were infected with the virus.	X5	0.735	0.9	0.839	0.919
I am worried that foreigners are spreading the virus because they’re not as clean as we are.	X6	0.509	0.848	0.670	0.911
**Contamination**			**0.914**		**0.897**
I am worried that if I touched something in a public space (e.g., handrail, door handle), I would catch the virus.	C1	0.784	0.891	0.783	0.878
I am worried that if someone coughed or sneezed near me, I would catch the virus.	C2	0.831	0.896	0.804	0.875
I am worried that people around me will infect me with the virus.	C3	0.708	0.895	0.692	0.884
I am worried about taking changes in cash transactions.	C4	0.675	0.907	0.714	0.879
I am worried that I might catch the virus from handling money or using a debit machine.	C5	0.655	0.887	0.670	0.868
I am worried that my mail has been contaminated by mail handlers.	C6	0.473	0.913	0.702	0.888
**Traumatic Stress**			**0.844**		**0.929**
I had trouble concentrating because I kept thinking about the virus.	T1	0.494	0.801	0.553	0.917
Disturbing mental images about the virus popped into my mind against my will.	T2	0.424	0.806	0.588	0.923
I had trouble sleeping because I worried about the virus.	T3	0.298	0.815	0.428	0.91
I thought about the virus when I didn’t mean to.	T4	0.509	0.818	0.715	0.911
Reminders of the virus caused me to have physical reactions, such as sweating or a pounding heart.	T5	0.305	0.839	0.787	0.92
I had bad dreams about the virus.	T6	0.248	0.828	0.665	0.918
**Compulsive Checking**			**0.793**		**0.783**
Searched the Internet for treatments for COVID-19.	CH1	0.557	0.738	0.876	0.74
Asked health professionals (e.g., doctors or pharmacists) for advice about COVID-19.	CH2	0.650	0.735	0.848	0.752
Checked YouTube videos about COVID-19.	CH3	0.211	0.788	0.868	0.751
Checked your own body for signs of infection (e.g., taking your temperature).	CH4	0.917	0.741	0.649	0.745
Sought reassurance from friends or family about COVID-19.	CH5	0.671	0.763	0.697	0.757
Checked social media posts concerning COVID-19.	CH6	0.331	0.791	0.563	0.757

D—danger; SEC—socio-economic consequences; X—xenophobia; C—contamination; T—traumatic stress; CH—compulsive checking. ^1^ The Cronbach’s Alpha Values in Each Item Row Are the Cronbach’s Alpha Values Recalculated after Removing the Item from the Factor, while the Cronbach’s Alpha Values in the Factor Name Row Are the Values Calculated with the Six Items Together.

**Table 4:** Test results of the convergent validity of the CSS questionnaire obtained using the PSS and WBI-5 questionnaires.

	Representative Sample (n = 1200)		Student Sample (n = 350)	
	WBI-5	PSS	WBI-5	PSS
CSS-H total	−0.114 ***	0.112 ***	−0.199 ***	0.300 ***
CSS-H danger	−0.112 ***	0.145 ***	−0.170 ***	0.245 ***
CSS-H socio-economic consequences	−0.140 ***	0.191 ***	−0.145 ***	0.233 ***
CSS-H Xenophobia	−0.052	0.112 ***	−0.064	0.088
CSS-H contamination	−0.007	−0.003	−0.080	0.166 ***
CSS-H traumatic stress	−0.306 ***	0.121 ***	−0.201 ***	0.295 ***
CSS-H compulsive checking	−0.031	0.045	−0.135 **	0.175 ***

*** *p* < 0.01; ** *p* < 0.05.

## Data Availability

The dataset supporting the conclusions of this article is available from the corresponding author on reasonable request in accordance with MDPI Research Data Policies.

## Appendix A

**Table A1:** Items in the Hungarian version of the CSS questionnaire.

**Veszély (Danger)**	
Aggódom, hogy elkapom a vírust.	D1
Aggódom, hogy nem tudom megvédeni a családom a vírustól.	D2
Aggódom, hogy egészségügyi rendszerünk nem lesz képes megvédeni szeretteimet.	D3
Aggódom, hogy az egészségügyi rendszerünk nem képes megvédeni a vírustól.	D4
Aggódom, hogy az alapvető higiéniás szabályok betartása (pl. kézmosás) nem elegendők, hogy megvédjenek a vírustól.	D5
Aggódom, hogy a szociális távolságtartás nem elegendő, hogy megvédjen a vírustól.	D6
**Szocioökonómiai Következmények (Socio-Economic Concesquences)**	
Aggódom, hogy az élelmiszerüzletek kifogynak az élelmeből.	SE1
Aggódom, hogy az élelmiszerüzletek bezárnak.	SE2
Aggódom, hogy az élelmiszerüzletek kifogynak a tisztító és fertőtlenítő szerekből.	SE3
Aggódom, hogy a boltokból elfogynak a megfázás és nátha elleni gyógyszerek.	SE4
Aggódom, hogy az üzletek kifogynak az ivóvízből.	SE5
Aggódom, hogy a gyógyszertárak kifogynak a vényköteles gyógyszerekből.	SE6
**Idegenellenesség (Xenophobia)**	
Aggódom, hogy a külföldiek terjesztik a vírust hazánkban.	X1
Ha külföldi ételeket kínáló étterembe megyek, akkor aggódom, hogy elkapom a vírust.	X2
Aggódom, hogy külföldiekkel kapcsolatba kerüljek, mert ők hordozhatják a vírust.	X3
Ha találkozom egy külföldivel, akkor aggódni kezdek, hogy ő esetleg hordozza vírust.	X4
Ha egy liftben lennék egy csoport külföldivel, aggódnék, hogy ők vírussal fertőzöttek.	X5
Aggódom, hogy a külföldiek terjesztik a vírust, mert nem olyan tiszták/higiénikusak, mint mi.	X6
**Megfertőződés (Contamination)**	
Aggódom, hogy ha hozzáérek valamihez nyilvános helyen (pl. korláthoz, kilincshez), akkor elkaphatom a vírust.	C1
Aggódom, hogy ha valaki köhög vagy tüsszent a közelemben akkor elkapom a vírust.	C2
Aggódom, hogy a környezetemben lévő emberek megfertőznek a vírussal.	C3
Aggódom a készpénzes fizetés miatt.	C4
Aggódom, hogy elkaphatom a vírust, ha pénzhez nyúlok vagy bank automatát használok.	C5
Aggódom, hogy a leveleim beszennyeződnek/fertőződnek a postás által.	C6
**Traumatikus Stressz (Traumatic Stress)**	
Nehezen tudtam koncentrálni, mert folyamatosan a vírusra gondoltam.	T1
Akaratom ellenére a vírussal kapcsolatos nyugtalanító képek ugrottak be.	T2
Alvási problémáim voltak a vírussal kapcsolatos aggodalmaim miatt.	T3
Akkor is a vírusra gondoltam, amikor nem akartam.	T4
A vírussal kapcsolatos figyelemfelhívó tájékoztatók fizikai tüneteket váltottak ki belőlem, úgy mint izzadás vagy szívdobogás érzés.	T5
A vírussal kapcsolatos rossz álmaim voltak.	T6
**Megszállott Keresés (Compulsive Checking)**	
Kutattam az interneten a COVID-19 gyógymódjai után.	CH1
Tanácsot kértem egészségügyi szakemberektől (pl. orvostól, gyógyszerésztől) a COVID-19-el kapcsolatban.	CH2
COVID-19-el kapcsolatos YouTube videókat néztem.	CH3
Ellenőriztem a testemet a fertőzés jeleit keresve (pl. megmértem a lázamat).	CH4
Megnyugtatást kértem a családomtól és a barátaimtól a COVID-19-miatt.	CH5
A COVID-19-re vonatkozó posztokat követtem a közösségi médiában.	CH6
